# Brazilian Green Propolis Inhibits Inflammatory Angiogenesis in a Murine Sponge Model

**DOI:** 10.1093/ecam/nep197

**Published:** 2011-03-09

**Authors:** Sandra Aparecida Lima de Moura, Mônica Alves Neves Diniz Ferreira, Silvia Passos Andrade, Maria Leticia Costa Reis, Maria de Lourdes Noviello, Denise Carmona Cara

**Affiliations:** ^1^Department of General Pathology, Institute of Biological Sciences, Federal University of Minas Gerais (UFMG), Belo Horizonte, Brazil; ^2^Department of Physiology and Biophysics, Institute of Biological Sciences, Federal University of Minas Gerais (UFMG), Belo Horizonte, Brazil

## Abstract

Angiogenesis and inflammation are persistent features of several pathological conditions. Propolis, a sticky material that honeybees collect from living plants, has been reported to have multiple biological effects including anti-inflammatory and anti-neoplasic activities. Here, we investigated the effects of water extract of green propolis (WEP) on angiogenesis, inflammatory cell accumulation and endogenous production of cytokines in sponge implants of mice over a 14-day period. Blood vessel formation as assessed by hemoglobin content and by morphometric analysis of the implants was reduced by WEP (500 mg kg^−1^ orally) compared to the untreated group. The levels of vascular endothelial growth factor (VEGF) increased progressively in the treated group but decreased after Day 10 in the control group. Accumulation of neutrophils and macrophages was determined by measuring myeloperoxidase (MPO) and *N*-acetyl-*β*-_D_-glucosaminidase (NAG) activities, respectively. Neutrophil accumulation was unaffected by propolis, but NAG activity was reduced by the treatment at Day 14. The levels TGF-**β**1 intra-implant increased progressively in both groups but were higher (40%) at Day 14 in the control implants. The pro-inflammatory levels of TNF-**α** peaked at Day 7 in the control implants, and at Day 14 in the propolis-treated group. Our results indicate that the anti-inflammatory/anti-angiogenic effects of propolis are associated with cytokine modulation.

## 1. Introduction

Compelling evidence indicates that angiogenesis and inflammation are key components to the maintenance of a variety of pathological conditions such as rheumatoid arthritis, psoriasis, atherosclerosis, neoplasias and Crohn's disease. Both processes are mediated by a number of molecules and cellular events that are essential for activation of leukocytes and formation of new blood vessels. These include vascular endothelial growth factor (VEGF), tumor necrosis factors (TNF) and transforming growth factors (TGF). Thus, attenuation and/or inhibition of blood vessel formation and inflammatory cell influx to the sites of the injuries would represent a way to control pathophysiological processes.

Propolis, a substance produced by honeybees, has a very complex chemical composition and is dependent upon the source plant [1]. It has been found to exhibit anti-bacteria, anti-inflammatory, anti-oxidant and anti-tumor activities [1–6]. These effects of propolis have been attributed to its main components particularly artepillin C (a predominant metabolite from green Brazilian propolis) and caffeic acid phenethyl ester (a metabolite found in Brazilian green propolis and in propolis from temperate regions) [7–9]. Furthermore, propolis extract has been shown to suppress corneal neovascularization in rabbit's cornea injured by silver nitrate cauterization, through cyclo- and lipooxygenase pathways [10], and to inhibit angiogenesis in the chick embryo chorioallantoic membrane [11]. Studies *in vitro* using human umbilical vein endothelial cells and calf pulmonary arterial endothelial cells have shown that propolis extract inhibited cell proliferation, migration and capillary tube formation in a dose-dependent manner [11, 12].

Considering the above-mentioned effects of propolis and that angiogenesis and inflammation co-exist in a variety of pathological conditions, we hypothesized that propolis might prevent inflammatory angiogenesis. To our knowledge, there is no study in which the effects of water extract of green propolis (WEP) on these processes have been performed simultaneously in a model of inflammatory angiogenesis. The murine sponge model of angiogenesis offers a unique opportunity to explore key components of these mutually co-dependent processes allowing the characterization of both acute and chronic inflammatory responses as well as key components of fibrovascular tissue (cell influx, blood vessel formation and extracellular matrix deposition). The effects of various anti-inflammatory/anti-angiogenic compounds have also been determined using this implantation technique [13, 14].

The present study explores the hypothesis that WEP may prevent inflammatory angiogenesis in the sponge implant model by determining the effects of systemic administration of this compound on neovascularization, accumulation of neutrophils and macrophages together with relevant pro-angiogenic and pro-inflammatory cytokines.

## 2. Methods

### 2.1. Preparation of the Aqueous Extract of Green Propolis

Sample of Brazilian green propolis was collected in Jaguaraçu/Minas Gerais state, Brazil (September 2005 to September 2006) from *Apis mellifera* hives. The sample was homogenized and frozen at −18°C and an aliquot (200 g) was crushed and 500 ml of distilled water added to make a suspension. This suspension was maintained for a period of 30–60 min under stirring with a temperature of 70°C until full incorporation of propolis. The material was filtered through paper Whatman #1 to obtain the first fraction of the extract and waste. The residue received the same treatment to obtain the second fraction of the extract. The two fractions of the extract were added together and then lyophilized. For administration to the animals, the compound was diluted in water and 200 *μ*l of the suspension containing 15 mg (500 mg kg^−1^) of the extract was given to each animal.

### 2.2. Animals

Female Swiss mice 7-8 weeks (30–35 g body weight) used in these experiments were provided by the Central Animal Facility at the Institute of Biological Sciences, Federal University of Minas Gerais, Brazil. The animals were housed individually and provided with chow pellets and water *ad libitum*. The light/dark cycle was 12:12 h with lights on at 7:00 a.m. and lights off at 7:00 p.m. Efforts were made to avoid all unnecessary distress to the animals. Housing, anesthesia and post-operative care adhered with the guidelines established in accordance to the Federal University of Minas Gerais Guidelines for Animal Experimentation. This experiment was approved by the University's Committee for Animal Experimentation (Protocol 161/2005).

### 2.3. Preparation of Sponge Disks, Implantation and Treatment

Polyether-polyurethane sponge (Vitafoam Ltd, Manchester, UK) was used as the implanted material. The implants were diskshaped, 5 mm thick × 8 mm diameter and were soaked overnight in 70% v/v ethanol and sterilized by boiling in distilled water for 15 min before implantation. Mice were anesthetized by intra-peritoneal injection of 4 *μ*l g^−1^ of a mixture of ketamine (150 mg kg^−1^) and xylazine (10 mg kg^−1^), their dorsal hair was shaved and their skin was wiped with 70% ethanol in preparation for implantation. The sponge disks were aseptically implanted into a subcutaneous pouch, which had been made with curved artery forceps through a 1 cm long dorsal mid-line incision. Post-operatively, the animals were monitored for any signs of infection at the operative site, discomfort or distress; any showing such signs were immediately killed. Water extract of green propolis (WEP) was given orally by gavage to provide 500 mg kg^−1^ day^−1^. The treatment started on the day of the implantation and lasted for 14 days. The control group of mice received saline on the same schedule. The dose of the compound was chosen based on the pilot experiments and on data from the literature [15, 16]. The treatment and sponge implants were well tolerated by the mice during the experimental period.

### 2.4. Hemoglobin Extraction

The extent of the vascularization of the sponge implants was assessed by the amount of hemoglobin (Hb) detected in the tissue using the Drabkin method [17]. At Days 4, 7 and 14 post-implantation, groups of animals were euthanized and the sponge implants carefully removed. The sponges were dissected from adherent tissue, weighed, homogenized (Tekmar TR-10, OH) in 2 ml of Drabkin reagent (Labtest, São Paulo, Brazil) and centrifuged at 4°C at 12 000 g for 20 min. The supernatants were filtered through a 0.22 *μ*m Millipore filter. The hemoglobin concentration of the samples was determined spectrophotometrically by measuring absorbance at 540 nm using an ELISA plate reader and was compared against a standard curve of hemoglobin. The content of hemoglobin in the implant was expressed as micrograms of Hb per milligram wet tissue.

### 2.5. Tissue Extraction and Determination of Myeloperoxidase and *N*-Acetyl-*β*-_D_-Glucosaminidase Activities

The extent of neutrophil accumulation in the implants was measured by assaying myeloperoxidase (MPO) activity as previously described [18]. After processing the supernatant of the implants for the hemoglobin determination (see above), a part of the corresponding pellet was weighed, homogenized in (2 ml) pH 4.7 buffer (0.1 M NaCl, 0.02 M Na_3_PO_4_, 0.015 M Na_2_-EDTA) and centrifuged at 4°C at 12 000 g for 10 min. The pellets were then re-suspended in 0.05 M sodium phosphate buffer (pH 5.4) containing 0.5% hexa-1,6-*bis*-decyltrimethylammonium bromide (HTAB). MPO activity in the supernatant samples was assayed by measuring the change in absorbance (optical density; OD) at 450 nm using 3,3′-5,5′-tetramethylbenzidine (TMB) prepared in dimethyl sulfoxide (DMSO) in a final concentration of 1.6 mM and H_2_O_2_ (0.3 mM) in the sodium phosphate buffer, pH 6.0. The reaction was terminated by the addition of 50 *μ*l of H_2_SO_4_ (4 M). Results were expressed as change in OD per mg of wet tissue.

The infiltration of mononuclear cells into the implants was quantified by measuring the levels of the lysosomal enzyme *N*-acetyl-*β*-d-glucosaminidase (NAG) which is present in high levels in activated macrophages [18]. Part of the pellet that remained after the hemoglobin measurement was kept for this assay. These pellets were weighed, homogenized in NaCl solution (0.9% w/v) containing 0.1% v/v Triton X-100 (Promega) and centrifuged (3000 g; 10 min at 4°C). Samples of the resulting supernatant (100 *μ*l) were incubated for 10 min with 100 *μ*l *p*-nitrophenyl-*N*-acetyl-*β*-d-glucosaminide (Sigma) prepared in the citrate/sodium phosphate buffer (0.1 M citric acid, 0.1 M Na_2_HPO_4_; pH 4.5) to yield a final concentration of 2.24 mM. The reaction was stopped by the addition of 100 *μ*l of 0.2 M glycine buffer (pH 10.6). Hydrolysis of the substrate was determined by measuring the absorption at 400 nm. NAG activity was expressed as the change in OD per milligram of wet tissue (implant).

### 2.6. VEGF, TNF-*α* and TGF-*β*1 Production in the Sponge Implants

For this procedure, the implants were removed at Days 4, 7 and 14 post-implantation, homogenized in 1.0 ml sodium–phosphate buffered saline (PBS) pH 7.4 containing 0.05% Tween-20 and centrifuged at 4°C for 10 min at 10 000 g. The cytokines in the supernatant from each implant were measured in 50 *μ*l of the supernatant using Immunoassay ELISA Kits (R&D Systems, USA) for murine VEGF, TNF-*α* and TGF-*β*1 following the manufacturer's protocol. Briefly, flat-bottom, 96-well microtiter plates were coated with a specific murine monoclonal antibody against the cytokine for 18 h at 4°C and then washed with PBS (pH 7.4) containing 0.05% Tween-20 (wash buffer). Non-specific binding sites were blocked with 1% bovine serum albumin (BSA) in PBS (blocking buffer). Plates were rinsed with the wash buffer and samples (in 0.1% BSA in PBS-reagent diluent) were added to the wells, followed by incubation for 18 h at 4°C.

Plates were then washed and appropriate second horseradish peroxidase-conjugated polyclonal antibody against the cytokine was added to the wells. Plates were then washed and the chromogen substrate OPD (*o*-phenylendiamine, Sigma) diluted in 0.03 M citrate buffer (pH 5.0) containing 0.02% 30 v/v H_2_O_2_ was added. The plates were incubated in the dark for 30 min at room temperature. The reaction was stopped with 2 N sulfuric acid solution (50 *μ*l/well). The intensity of the color was measured at 492 nm on a spectrophotometer (Emax-Molecular Devices). Standards were 0.5-log_10_ dilutions of recombinant murine cytokines from 7.5 to 1000 pg ml^−1^ (100 *μ*l). The results were expressed as picograms of cytokine per milligram wet tissue.

### 2.7. Morphometric Analysis and Blood Vessel Quantification

To examine the degree of neovascularization in the implants of control (saline) and treated mice, a total of 18 sponge disks (three for each time point for each group) were processed for histological assessment. The implants were harvested, embedded in paraffin and 5-*μ*m-thick sections and stained with hematoxylin and eosin (HE) and examined under a light microscope. Microscopic images of cross-sections (5 *μ*m) were obtained with a planapochromatic objective 40x in light microscopy. The images were digitized through a JVC TK-1270/JGB microcamera and transferred to an image analyzer (Kontron Eletronics, Carl Zeiss-KS300 version 2). A countable microvessel was defined as a structure with a lumen that contained red blood cells or not. To establish the minimal representative microscopic fields per sample, blood vessels were counted in 50 fields (400x magnification, that is, 40 objective lens and 10 ocular lens; 0.053 mm^2^ per field) from a randomly chosen slide in each group as described previously [19]. The results were expressed as mean  ±  SEM of the total number of vessels/15 fields.

### 2.8. Statistical Analysis

Results are presented as mean  ±  SEM. Statistical comparisons between two groups (*N* = 8–10 per group) of mice were carried out using Student's *t*-test for unpaired data. A *P* < .05 was considered to be statistically significant. Statistical analysis was performed using Graph-Pad Prism 4.01.

## 3. Results

### 3.1. General Assessment of Systemic Administration of Propolis

Systemic administration of propolis (500 mg kg^−1^ day^−1^) during 14 days showed no signs of toxicity such as weight loss, sedation or alterations in motor activity of the animals. The surgical procedure, the sponge matrix and the treatment were well tolerated by all animals. No signs of infection or rejection were observed in the implant compartment during the 14-day period of the experiment.

## 4. Anti-Angiogenic Effects

**Figure 1:**
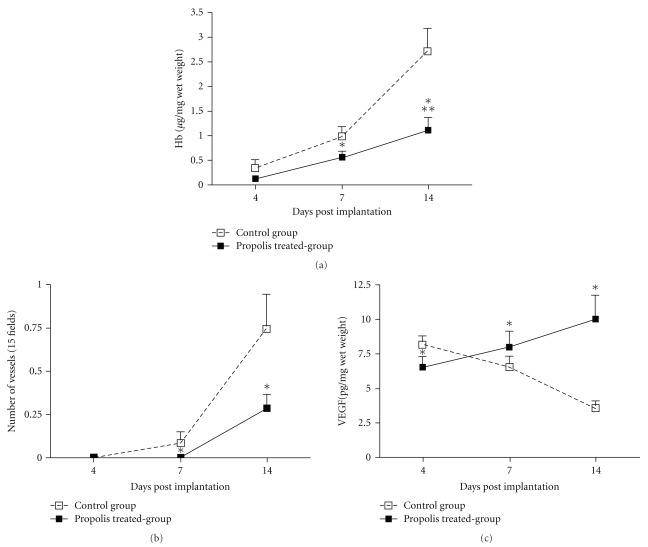
Effects of WEP on angiogenesis in sponge implants. The hemoglobin content after the systemic treatment (500 mg kg^−1^ day^−1^) relative to the control group (a). Morphometric analysis of the vessel number and the levels of VEGF are shown in (b) and (c), respectively. Values are the means (±SEM) from groups of 8–10 animals. *P < .05 versus the control group.

Daily oral doses of WEP (500 mg kg^−1^) given by gavage to different groups of mice over 14 days reduced neovascularization of the implants, as detected by changes in the hemoglobin content. A decrease in hemoglobin content was observed at Days 7 and 14 after the treatment with WEP (Figure 1(a)). This difference was further confirmed by the morphometric analysis of the implants showing that the number of vessels was markedly lower in the treated group compared with the control group (Figure 1(b)). 

Because VEGF has been considered a marker for angiogenesis, we have assayed for this molecule in the implants. Interestingly, the levels of this cytokine increased progressively in the propolis-treated group but decreased in the control group (Figure 1(c)).

### 4.1. Leukocyte Accumulation and Cytokine Production

**Figure 2:**
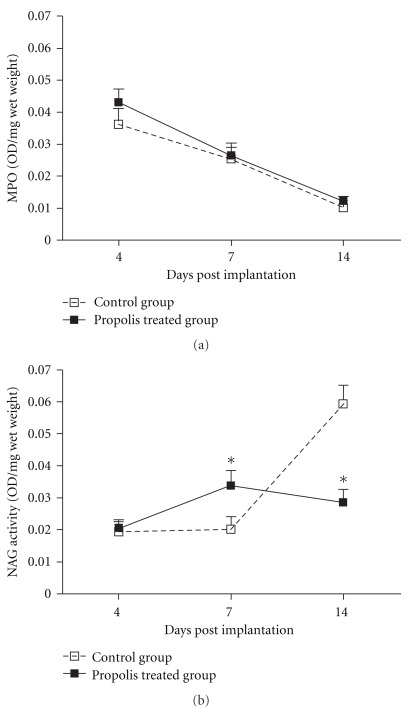
Effects of WEP (500 mg kg^−1^ day^−1^) on inflammatory enzyme activities. The level of neutrophil accumulation (measured as myeloperoxidase—MPO activity) is shown in (a). Macrophage accumulation (measured as *N*-acetyl-*β*-d-glucosaminidase-NAG activity) is shown in (b). Values are the means (±SEM) from groups of 8–10 animals in each group. *P < .05 versus the control group.

**Figure 3:**
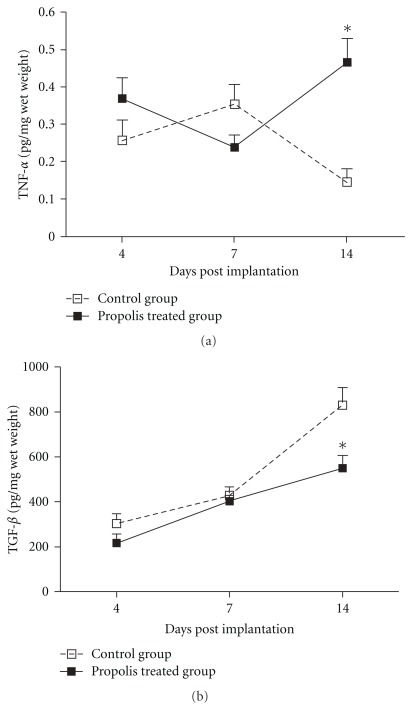
Effects of WEP (500 mg kg^−1^ day^−1^) on levels of TNF-*α* (a) and TGF-*β*1 (b). A distinct pattern in the production of these cytokines is observed between control and treated implants. Values shown are the means (±SEM) from groups of 8–10 animals. *P < .05 versus the control group.

The inflammatory components of the sponge-induced inflammation were determined by estimating the numbers of leukocytes in the implant through assays for enzyme activities. Neutrophil numbers (as MPO activity) was not affected by propolis treatment at the time points studied; however, macrophage accumulation (as NAG activity) decreased (*∼*50%) at Day 14 in the treated group compared with the control (Figures 2(a) and 2(b)). The levels of pro-inflammatory cytokine (TNF-*α*) peaked at Day 7 in the control group and at Day 14 in the propolis-treated group (Figure 3(a)). TGF-*β*1 levels increased progressively in both types of implant but propolis treatment was able to reduce the amount of this cytokine at Day 14 post-implantation (Figure 3(b)). 

## 5. Discussion

Propolis has been used for various medical purposes since antiquity, mainly as an anti-inflammatory agent and wound healing promoter [20]. In more recent times, there has been a considerable interest in the therapeutic properties of propolis as its anti-infectious, immunomodulatory and anti-neoplasic actions have been demonstrated [5, 21, 22]. Other actions of propolis assessed in experimental animals have proved beneficial on acute and chronic inflammation [23–25] and in inhibiting angiogenesis [10–12, 26].

Here, we investigated the effects of WEP on blood vessel formation, on inflammatory enzyme activities and on cytokines production in a model of sponge implant. Although, propolis extract has been shown to suppress corneal angiogenesis in rabbit's cornea injured by silver nitrate cauterization [10] and in the chick embryo chorioallantoic membrane [11, 12], our model of angiogenesis differs importantly from the above-mentioned angiogenesis systems particularly on its inflammatory component. The corneal model and chick choriallantoic membrane [27, 28] are minimal inflammatory systems compared to the other assays. In addition, to our knowledge, there is no report on the effects of WEP on key components of inflammatory angiogenesis (blood vessel formation, inflammatory enzyme activity and cytokine production). Thus, it seemed reasonable that this compound would modulate the host inflammatory response to synthetic implants. By assessing WEP in the sponge model, we were able to identify its effects on critical steps of the formation of the fibrovascular tissue. Our studies demonstrate a novel inhibitory activity of WEP on the inflammatory and angiogenic components of the newly formed tissue. The treatment also revealed the effects of WEP on pro-inflammatory and pro-fibrogenic cytokines within the sponge implants.

The sequential development of angiogenesis, inflammation and cytokine production in the fibrovascular tissue induced by sponge implantation in mice treated with WEP differed substantially from the untreated group. Angiogenesis assessed by the Hb content (vascular index) of the sponges and by morphometric analysis of blood vessel number increased progressively in both groups but much less in the propolis-treated group. Interestingly, the levels of the pro-angiogenic cytokine, VEGF, presented a different pattern of production in both groups. Thus, while in the propolis-treated group the levels increased progressively, in the control group the cytokine production decreased after Day 7 post-implantation. This lack of correlation between the levels of VEGF and Hb content in the control group is fully compatible with the notion that in the absence of hypoxic and/or oxidative stress, the levels of VEGF would drop [29, 30]. It is possible that the blood supply (Hb) content and vessel number in the implant compartment from Day 7 onwards in the control group would be enough to comply with the metabolic demand at the site which in turn would result in inhibitory signal to VEGF production. Conversely, in the propolis-treated group decreased angiogenesis stimulated the production of VEGF to compensate for deficient blood supply in the implant compartment.

The inflammatory components of the implants were assessed by inflammatory enzymes and cytokines. In the propolis-treated group, MPO activity (representing activated neutrophils) was not affected by the treatment, showing a lack of WEP effect on this inflammatory cell population. The compound, however, was able to inhibit markedly NAG activity (representing activated macrophages/monocytes) at Day 14 post-implantation suggesting a degree of selectivity of propolis for this cell population. Our results are in line with those by Orsolic and Basic [31] that showed a decrease in macrophage recruitment in the peritoneal cavity in animals treated with aqueous extract of propolis.

The pattern of TNF-*α* and TGF-*β*1 production (two relevant inflammatory and fibrogenic cytokines) was also influenced by WEP. The levels of TNF-*α* peaked in the control group at Day 7 and in the propolis-treated group at Day 14. Differently, the kinetics of TGF-*β*1 production in the implants had parallel courses in both types of implants but the amount of this cytokine was *∼*1.5-fold in the control group at Day 14 relative to the treated group. These results clearly indicate that the beneficial effects of propolis in our system have occurred through modulation of these cytokines that perform well-established roles in regulating extracellular matrix deposition in wound healing [32]. The fact that CAPE and other caffeoylquinic acids and artepillin C have been shown to exert most of the biological activities studied [3, 14, 23–25] and that these components were identified in our aqueous extract of the Brazilian green propolis [33], it seems reasonable to attribute, at least in part, the anti-inflammatory and anti-angiogenic effects of propolis to these components. We cannot rule out, however, the possibility that other constituents found in the green propolis are involved in the inhibition of key components of inflammatory angiogenesis observed in our study.

**Figure 4:**
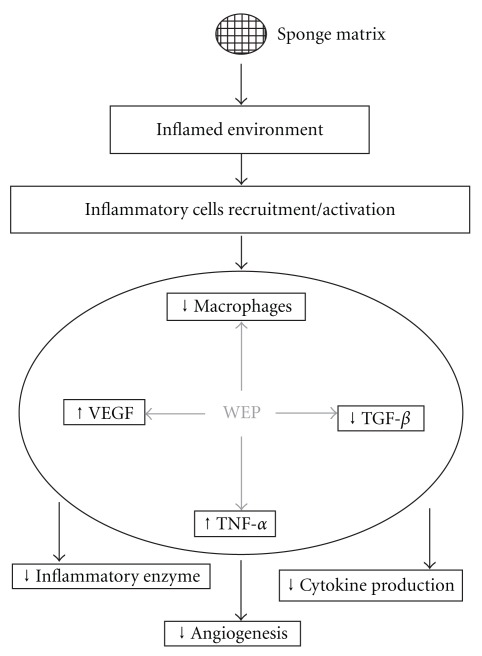
Diagram representing the sequence of major cellular and molecular events modulated by WEP in sponge-induced inflammatory angiogenesis. The effect of WEP decreasing macrophage recruitment/activation leads to low production of *N-*acety-*β*-_D_-glucosaminidase enzyme. WEP modulates cytokine production in the implants by increasing the levels of VEGF and TNF-*α* and decreasing the levels of TGF-*β*1. These cytokines play an important role in regulating extracellular matrix deposition.

To our knowledge, this is the first report that assessed the effects of WEP on multiple parameters of the main components of inflammatory angiogenesis. Moreover, a regulatory function of WEP on pro-inflammatory and pro-fibrogenic cytokines production has been revealed (Figure 4). Altogether, our results confirm and extend previous findings of the therapeutic effects of propolis which are likely to be associated with regulation of cytokines that are secreted by inflammatory cells at the site of tissue injury. 

## Funding

This work was supported by Conselho Nacional de Desenvolvimento Científico e Tecnológico (CNPq) and Fundação de Amparo à Pesquisa do Estado de Minas Gerais (FAPEMIG).
